# Epidural Anesthesia in a Patient With Turner Syndrome: A Case Report

**DOI:** 10.7759/cureus.95993

**Published:** 2025-11-03

**Authors:** John Quentin Deckbar, Luis Garcia, Leighan Bye, Zachary Cohen, Nuo Yang

**Affiliations:** 1 Department of Anesthesia, Indiana University School of Medicine, Indianapolis, USA

**Keywords:** difficult epidural, labor epidural analgesia, neuraxial anesthsia, neuraxial ultrasound, turner’s syndrome

## Abstract

Turner syndrome (TS) is associated with short stature, cardiac abnormalities, and musculoskeletal deformity, which pose unique obstetric anesthesia challenges in the small proportion of TS patients who go through spontaneous or assisted pregnancy. There is very limited data available on the anesthetic management of these patients. This case report presents the management of a 32-year-old gravida 2 para 0 (G2P0010) patient with mosaic TS undergoing labor induction at 37 weeks of gestation. An indwelling epidural catheter placed under ultrasound guidance provided adequate labor analgesia with about one-tenth of the dosing compared to the general population and led to a successful vaginal delivery without complications. This case highlights the unique challenges presented by the TS patients to obstetric anesthesia, such as difficult epidural placement and unusual neuraxial dosing, and provides important insight into the safe anesthetic practice in this patient population.

## Introduction

Turner syndrome (TS) is a chromosomal disorder that affects approximately one in 2000-2500 live female births [1-3]. Of these cases, roughly 50% of patients will have pure monosomy (45, XO), whereas the remainder have some form of mosaicism or structural abnormality of the X chromosome [4]. This genetic disorder can involve several organ systems, particularly affecting cardiovascular, endocrine, reproductive, musculoskeletal, and neurological systems [3,4]. Reproductive abnormalities can begin during fetal development with germ cell apoptosis, which results in streak gonads and premature ovarian insufficiency [1,2]. Consequently, only 30% of patients undergo pubertal development to a certain extent, 10-20% achieve spontaneous menarche, and 2-8% experience spontaneous pregnancy, with about 60% of them resulting in live birth [1,2,5].

Pregnancy in TS patients carries increased maternal and fetal risks. First, cardiac abnormalities are considered the most serious medical problems associated with pregnant TS patients. Cardiovascular pathologies such as hypertension, coarctation of the aorta, bicuspid aortic valve, and ischemic heart disease predispose these patients to dire complications, including aortic dissection and heart failure [6-10]. Second, musculoskeletal abnormalities such as short stature and kyphoscoliosis [11] as well as higher body mass index (BMI) [12] commonly make the neuraxial placement challenging in these patients. Uneven distribution of neuraxial drugs is another concern [13]. Third, they are prone to have difficult airways due to micrognathia, short stature, webbed neck, and high arched palate [1,14]. Fourth, hepatic anomalies ranging from mild pathologies such as elevated liver enzymes and steatosis to more severe conditions such as cirrhosis and nodular regenerative hyperplasia, as well as renal abnormalities including horseshoe kidneys, malrotations, and hydronephrosis, may also make the metabolism of general anesthetics less predictable [3,4,6]. Last, pregnancy can be further complicated by higher obstetrical risks including miscarriage, placental insufficiency, preeclampsia, and fetopelvic disproportion [5,12]. Therefore, selection of an optimal obstetric anesthetic strategy can be convoluted in TS patients and should be based on thorough consideration and balancing of multiple factors, most importantly the cardiac pathologies, obstetric indications, hemodynamic instability, musculoskeletal changes, and airway management.

## Case presentation

A 32-year-old G2P0010 patient with a weight of 70 kg and a height of 143 cm (BMI of 34.1 kg/m^2^) presented for induction of labor at 37 weeks of gestation following a spontaneous pregnancy. She was karyotype confirmed with mosaic TS. She also had a history of chronic hypertension, hypothyroidism, gastroesophageal reflux disease, and miscarriage at 17 weeks of a previous spontaneous pregnancy. She was intubated previously for procedures under general anesthesia without complications. Pre-anesthetic evaluation revealed normal dentition, short neck, limited neck range of motion, and a Mallampati class III airway. Her transthoracic echocardiography revealed normal cardiac structure and function with an aortic root of 2.7 cm (aortic size index of 1.5 cm/m^2^). Her metabolic equivalents (METs) were above four. Obstetric imaging showed a lobulated uterus and occlusion of the left fallopian tube. Patient ate a full meal within one hour of her request for labor analgesia.

**Figure 1:**
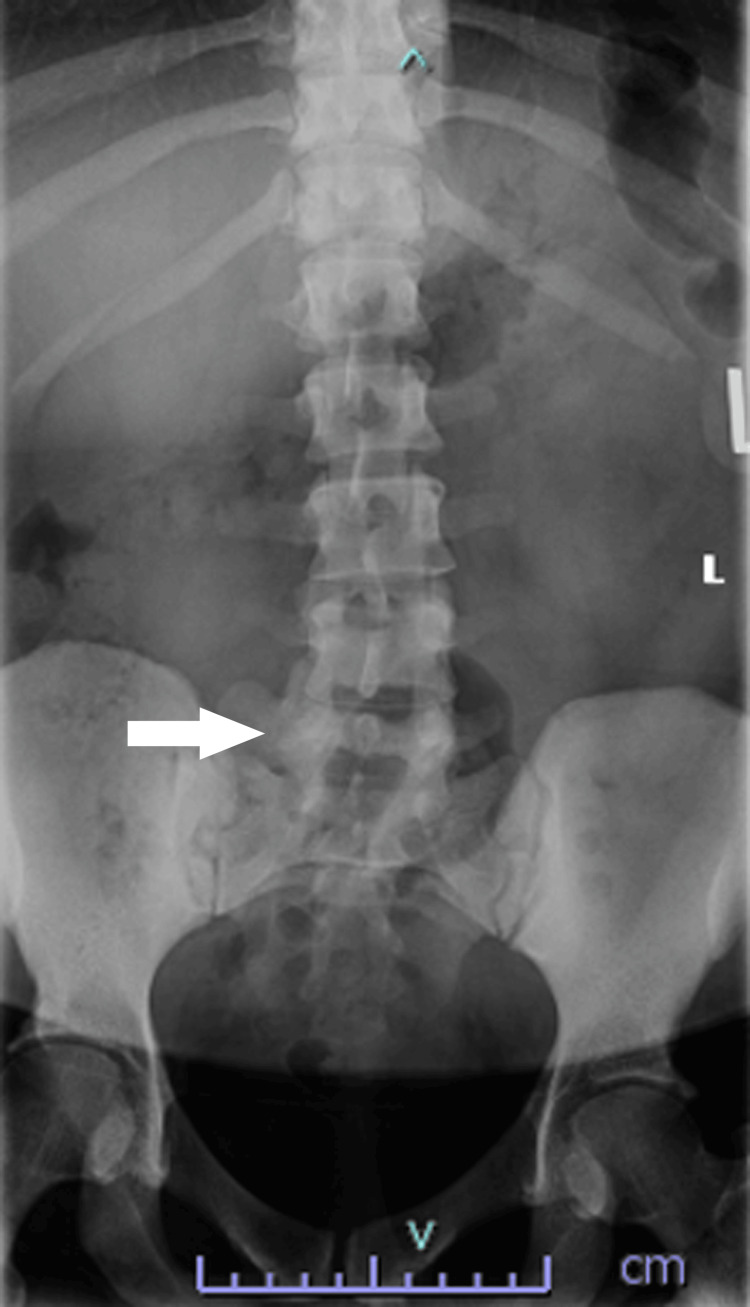
X-ray shows lumbar scoliosis in this patient. The arrow points to the lumbar scoliosis at her L4 level.

An extensive discussion between the anesthesiologist and obstetrician was initiated to comprehensively evaluate the associated risks and effectively communicate the planned anesthesia and procedure to the patient. An anesthesiologist was also notified by the obstetric team that the patient had been having fetal decelerations that warranted closer monitoring. After thorough counseling, the parturient consented to labor epidural analgesia. The patient’s coagulation profile was within the normal limits, and we proceeded to attempt an epidural for labor analgesia. The patient was placed in a sitting position. The first attempt was made by palpation with a 17-gauge Tuohy needle into the proximal L4-5 intervertebral space via the midline approach. Loss of resistance with saline was achieved, but we failed to thread the catheter, most likely due to her narrowed intervertebral and epidural space, even though we expanded the space with about 8 ml of saline. Due to her lumbar scoliosis (Figure 1) and to reduce discomfort and hypertension caused by the procedure, we used ultrasound scanning to facilitate our second attempt. An indwelling epidural catheter was then successfully advanced through her L3-4 intervertebral space at the 10-cm mark, with the loss of resistance achieved at 5 cm from skin, with no blood or cerebrospinal fluid aspirated. A test dose of 3 mL of 1.5% lidocaine with 1:200,000 epinephrine was administered with no reactions appreciated.

Soon after, we lay the patient supine and, within 10 minutes after the test dose injection, she reported full labor analgesia with contractions confirmed on the monitor. Upon block assessment with the cold and pin-prick tests, a bilateral sensory block was achieved at her T4 level. We thus decided to take a cautious approach with close monitoring of her vital signs and postponing the start of the epidural drip until further assessment. About fifty minutes after the initial test dose, the patient reported a sensation of pain with contractions again. We then administered a 1 mL bolus of the epidural drip solution composed of 0.1% bupivacaine with 2 mcg/mL fentanyl. The patient reported fast analgesia again. Another 1 mL of the same bolus was administered into her epidural catheter one hour later when she reported contraction pain again, followed by a continuous infusion of 1 mL/hour, which was significantly lower than our standard rate of 8-12 mL/hour. Her hemodynamics remained stable with systolic blood pressure in the range of 120s mmHg, diastolic blood pressure in the 70s mmHg, heart rate in the 90s bpm, and pulse oximeter readings of 96-99% on room air, and fetal monitoring was reassuring. The patient soon entered stage 2 of labor, and her epidural drip was thus discontinued. The total dose given to this patient was 3.37 mL until she vaginally delivered a 2100-g female baby with no complications. The patient was discharged home two days later. She and her baby kept doing well with breastfeeding and lack of complications on her postpartum visit one month later.

## Discussion

Managing anesthesia in obstetric TS patients demands careful consideration of various risk factors and a delicate balance to achieve safe and effective pain relief due to their unique clinical presentations. Except for a case report on the elective cesarean delivery using combined spinal-epidural (CSE) anesthesia in a TS patient [15], to our knowledge, we present the first case report of labor epidural analgesia in a TS patient with a positive outcome. This case highlights some important points on the anesthetic management in this unique patient population.

Deciding between general and regional anesthesia can be challenging [13-15]. All risk factors, including hemodynamic instability inflicted by intubation and neuraxial anesthesia, as well as difficult airway management and difficult neuraxial placement, have to be delicately balanced. As mentioned before, TS is associated with cardiovascular pathologies such as hypertension, coarctation of the aorta, bicuspid aortic valve, and ischemic heart disease [6-10]. These patients also often have a short webbed neck with reduced mobility as well as maxillary and mandibular hypoplasia [1,14], making them at higher risk for difficult airway management. Taken together, a multi-disciplinary approach to the care of these complex patients must include priority towards the most significant pathologies. For example, neuraxial anesthesia may be too time-consuming and completely impractical in an emergency, but would be preferred for elective procedures given the high risk for a difficult airway. In general, neuraxial anesthesia is favored secondary to its avoidance of difficult airway management and sympathetic surge associated with endotracheal intubation that may cause aortic dissection. Neuraxial placement tends to be challenging in TS patients due to their commonly seen short stature and skeletal abnormalities, which can be facilitated with ultrasound guidance, as we did in this case.

We only used about one-tenth of the standard epidural dosing in this patient and achieved adequate analgesic effect, which highlights the need for extreme caution with neuraxial dosing in TS patients. Difficulty in identifying the subarachnoid or epidural space, increased risk of inadvertent dural puncture, as well as uneven distribution and sensitivity to neuraxial drugs have been noted in TS patients [13], which may all contribute to the unusual dosing in our patient, in addition to her short stature and scoliosis. Although the lack of aspiration of cerebrospinal fluid and lack of signs and symptoms of postdural puncture headache make the intrathecal placement less likely, it still exists as a possibility, as well as an accidental dural tear. CSE anesthesia was used successfully for an elective cesarean delivery in a TS patient by Kalopita et al [15]. Although we performed epidural anesthesia to avoid possible exacerbation of fetal decelerations from CSE [16], in retrospect, CSE or dural puncture epidural (DPE) may be the preferred neuraxial strategy in TS patients without cardiac pathologies, as it can provide a definitive confirmation of the catheter placement. However, dural puncture with CSE or DPE may result in similar dosing problems as we encountered here. Of important note, the habitus of our TS patient was much more challenging compared to the patient in the other case report [15], with a height of 143 cm versus 155 cm and a BMI of 34 versus 23, which definitely contributed to the increased difficulty with neuraxial anesthesia placement and dosing. In summary, neuraxial anesthesia is generally preferred for TS parturients. In addition, thorough pre-anesthetic evaluation, assistance with ultrasound imaging, and extreme caution with neuraxial dosing should be used for patient safety.

## Conclusions

Anesthesiologists are faced with difficult challenges and dilemmas when managing obstetric TS patients due to their unique clinical features. Our case report provides an example of successful labor epidural analgesia. It highlights the decision-making process, technical challenges with neuraxial placement that may be overcome by ultrasound guidance, as well as careful and personalized neuraxial anesthetic dosing in this patient population.
